# Household food insecurity and physical activity behaviour in Ecuadorian children and adolescents: findings from the Ecuador 2018 National Health and Nutrition Survey (ENSANUT-2018)

**DOI:** 10.1017/S1368980024000351

**Published:** 2024-02-02

**Authors:** Rishika Chakraborty, Rodrigo X Armijos, M Margaret Weigel

**Affiliations:** 1 Department of Environmental and Occupational Health, Indiana University-Bloomington School of Public Health, 1025 E 7th St, Bloomington, IN 47405, USA; 2 Global Environmental Health Research Laboratory, Indiana University-Bloomington School of Public Health, Bloomington, IN, USA; 3 Center for Latin American & Caribbean Studies, Indiana University-Bloomington, Bloomington, IN, USA; 4 IU Center for Global Health, Indiana University-Bloomington, Bloomington, IN, USA

**Keywords:** Household food insecurity, Physical activity, Sedentary behaviour, Children and adolescents, Ecuador

## Abstract

**Objective:**

Ecuador has a high prevalence of household food insecurity (HFI) and is undergoing nutritional and epidemiologic transition. Evidence from high-income countries has reported negative or null associations between HFI and physical activity (PA) in children. It remains uncertain whether the same is true of those from low- and middle-income countries like Ecuador whose environmental and socio-demographic characteristics are distinct from those of high-income countries. We aimed to investigate the association of HFI with PA, sedentary behaviour (SB) and anthropometric indicators in children.

**Design:**

Cross-sectional analysis of data from the nationally representative 2018 Ecuadorian National Health and Nutrition Survey. Data were collected on HFI, PA, SB, socio-demographic characteristics and measured height and weight. Unadjusted and adjusted linear, log-binomial and multinomial logistic regression analyses assessed the relationship of HFI with PA, SB, stunting and BMI-for-age.

**Setting:**

Ecuador.

**Participants:**

23 621 children aged 5–17 years.

**Results:**

Marginal and moderate-severe HFI was prevalent in 24 % and 20 % of the households, respectively. HFI was not associated with PA, SB, stunting nor underweight. Moderate-severe HFI was associated with a lower odds of overweight and obesity. However, adjustment for household assets attenuated this finding for overweight (adjusted OR:0·90, 95 % CI: 0·77, 1·05) and obesity (adjusted OR: 0·88, 95 % CI: 0·71, 1·08).

**Conclusion:**

HFI is a burden in Ecuadorian households, but is not associated with PA, SB nor anthropometric indicators in children aged 5–17 years. However, a concerning prevalence of insufficient PA was reported, emphasising the critical need for evidence-based interventions aimed at promoting PA and reducing SB.

Physical activity (PA) is well documented to provide important benefits to child and adolescent health and well-being including reducing the risk for overweight and obesity, cardiometabolic risk factors and mental health as well as improved quality of life^(1)^. Guidelines established in 2010 by the WHO recommend that children aged 5–17 years old should perform at least 60 min of moderate to vigorous intensity PA daily^(2)^. However, globally, in 2016, four out of five school children between the ages 11 and 17 were estimated to engage in insufficient PA^(3)^. Between 2007 and 2013, only about 15 % of adolescents met PA recommendations in Latin American and Caribbean countries^(4)^.

Emerging evidence suggests PA levels in children may be associated with food insecurity. Household food insecurity (HFI) is defined as the limited or uncertain availability of nutritionally adequate and safe foods and the limited or uncertain ability to acquire acceptable foods in socially acceptable ways^(5)^. Four studies carried out in the USA and other high-income countries identified an inverse relationship between food insecurity and PA^(6–9)^, while another in the same setting reported null findings^(10)^. A study in Brazil, an upper middle-income country, reported a lack of association between food insecurity and PA in adolescents^(11)^. More recently, two studies used pooled cross-sectional data from low-, middle- and high-income countries to assess the relationship between food insecurity and PA in children aged 11 years and older^(12,13)^. In one, the authors used data from ninety-five countries and found that food-insecure children had lower levels of PA compared with their food-secure counterparts^(13)^. The other study that used national survey data from eleven South American countries found that food-insecure adolescents were more likely to actively commute to school, participate in physical education classes and engage in less time sitting than food-secure adolescents, but did not find an association with total PA^(12)^. Therefore, existing literature is inconsistent with studies reporting positive, negative and null associations between HFI and PA.

Ecuador, like many other low- and middle-income countries (LMIC), is in the process of nutritional and epidemiologic transition, a population phase characterised by changes from traditional to western diets high in energy-dense foods, reductions in PA and a shift towards a more sedentary lifestyle, obesity and chronic degenerative diseases^(14)^. Several studies have reported low PA levels in Ecuadorian children^(3,4,15)^. Similar to the WHO recommendations, the Ecuadorian Ministries of Health and Education recommends at least 60 min of daily PA for children aged 5–17 years^(16)^. However, the 2018 Ecuador Report Card for PA found only about one in three children in this age group were physically active for at least 60 min per day^(17)^. Recent studies have also identified a high prevalence of overweight and obesity^(15)^, stunting^(14)^ and a double burden of malnutrition^(18)^, characterised by concomitant obesity and stunting in the same Ecuadorian households. Moreover, a high burden of Zn deficiencies and anaemia has been reported among children and adolescents in Ecuador^(14)^. In Ecuadorian school age children and reproductive age women, high rates of overweight/obesity co-exist with Zn deficiency or with anaemia^(14)^. This triple burden of micronutrient deficiencies, overweight/obesity and stunting could lead to fatigue, weakness and raise the risk of infectious illnesses, further disrupting child PA levels.

Ecuadorian households with children have a high prevalence of moderate-severe HFI^(19)^. HFI has been previously linked to poorer health outcomes in Ecuadorian children such as psychosocial dysfunction^(20)^, poor oral health^(21)^ and a dual burden of over- and under-nutrition^(18)^. Other studies have reported null associations between HFI and stunting^(22)^, overweight and obesity^(22–24)^. However, due to a lack of published studies, it remains uncertain whether HFI is associated with PA in children and adolescents in Ecuador. Considering the high prevalence of HFI and sub-optimal PA levels, Ecuadorian children may be at risk for overweight, obesity and cardiometabolic risk factors that can develop into comorbidities over the long term. Globally, prior studies have investigated these associations using different metrics and samples, which limits our ability to draw conclusions across studies. Thus, a better understanding of the relationship of HFI with PA in Ecuadorian children is crucial in identifying the most vulnerable groups that could potentially benefit from targeted interventions to alleviate HFI and prevent onset of chronic diseases.

Several potential pathways exist that might explain how HFI can influence child PA, sedentary behaviour (SB), stunting and overweight/obesity (Fig. 1). For example, food-insecure households may be forced to switch to cheaper, energy-dense, pro-inflammatory foods instead of more expensive but nutritious fruits, vegetables dairy and animal protein food^(23,25)^. This can not only affect the linear growth and weight gain patterns of children in those households but also adversely impact their micronutrient and protein status and immunocompetence. This situation can increase child risk for anaemia^(26)^, fatigue and infectious illnesses, which in turn can make them physiologically less able to engage in PA. In addition, the consumption of a pro-inflammatory diet has been linked to poorer cardiometabolic health and higher odds of metabolic syndrome in Ecuadorian school-age children^(27)^. Both undernutrition and overnutrition can potentially impact child PA levels. For instance, children who are stunted or underweight have a smaller body size, reduced muscle mass and expend lower total energy in PA^(28)^. However, children who are overweight/obese also may have lower PA levels compared with their normal weight counterparts, particularly in the case of activities involving runs and jumps^(28)^.

**Fig. 1:**
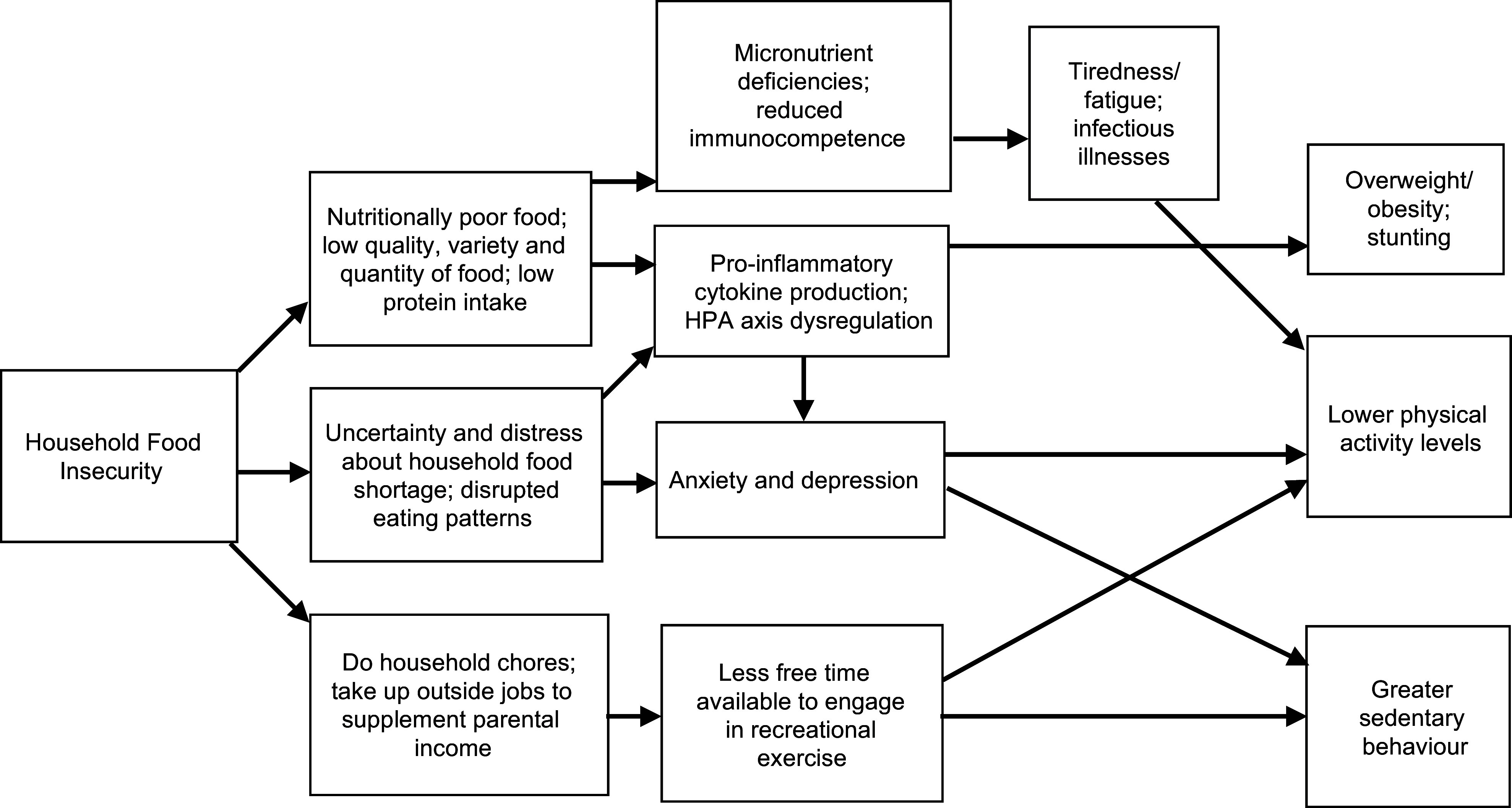
Potential pathways underlying the relationship between household food insecurity and physical activity, sedentary behaviour, overweight/obesity and stunting in Ecuadorian children and adolescents

Furthermore, children from food-insecure households are often aware of their household’s food situation^(29)^ and because of this may experience worry, sadness and anger^(29)^. Chronic stress due to worry or anxiety over the household food situation may also increase the production of pro-inflammatory cytokines and disrupt hypothalamic–pituitary–adrenal axis regulation^(30)^. Hypothalamic–pituitary–adrenal axis dysregulation promotes the desire to consume high sugar, energy-dense ‘comfort food’, that can lead to overweight/obesity^(30)^. Such disruption has also been associated with poorer mental health and well-being^(31)^. Stress and depression symptoms may further restrict child ability to participate in daily PA and may promote more SB^(9)^. Yet another potential pathway is that children from food-insecure households may be required to work outside the home to economically support their families or otherwise help out by performing domestic activities (e.g. household chores, sibling care)^(32)^. These may occupy time that they would have otherwise spent performing recreational PA.

The primary objective of this study was to investigate the association of HFI and PA and SB in 5–17-year-old children and adolescents from Ecuador, an upper-middle income country setting. We also assessed the relationship between HFI and anthropometric indicators of nutritional status, specifically stunting, underweight, overweight and obesity in these children. We hypothesised that children from food-insecure households would be more likely to have (a) lower PA levels, (b) greater SB and (c) present with stunting and overweight/obesity through one or more of the pathways described above.

## Methods

### Study Design and Sampling

We analysed cross-sectional data from the Ecuador 2018 National Health and Nutrition Survey (ENSANUT-2018).^(33)^ The study design, sampling strategy, data collection procedures and other methodological considerations used by the survey have been published in detail^(33)^. Briefly, the survey used a probabilistic two-stage sampling strategy to collect data from a nationally representative sample of 46 638 households of non-institutionalised individuals between 0 and 99 years of age. The the Ecuador 2018 National Health and Nutrition Survey collected data at the national, provincial, urban and rural levels during November 2018–January 2019 and June–July 2019. The underlying sampling frame for this survey was drawn from the 2010 Ecuadorian Population Census as well as the 2010–17 census updates^(33)^. We used data from two survey modules, the Household (‘Hogar’) and the Risk Factors (‘Factores de Riesgo’) for children 5–17 years old. These modules collected information on household socio-demographic characteristics, food security status, anthropometric measurements, PA and SB of children and adolescents, among other items. For the present study, our analytic sample was composed of all 23 621 children and adolescents included in the risk factors module who were aged 5–17 years.

### Study measures

#### Household food insecurity status

Food security status during the past 12 months was assessed using the eight-question Food Insecurity Experience Scale (FIES), Spanish language version, developed by the UN FAO^(34)^. The FIES was administered to adult caregivers, primarily mothers. The questions ranged from difficulties in accessing food due to lack of money or other resources to going without eating for a whole day. Affirmative responses were coded as ‘one’, while negative responses were coded as ‘zero’. The responses were summed to give a raw score ranging from zero to eight. Statistical validation of the FIES in this sample was conducted in a prior study^(35)^. Briefly, the eight-item FIES had infit values ranging from 0·86 and 1·17, residual correlations < 4 for all item pairs and a Rasch reliability of 0·75^(35)^. Therefore, this scale had adequate model fit and reliability^(35)^. In this study analysis, households with a raw score of zero were categorised as ‘food secure’, those with raw scores of 1–3 as ‘marginal HFI’ and scores of 4–8 as ‘moderate-severe HFI’.

#### Physical activity

The PA levels of the child participants were obtained by asking their caregivers three questions: (1) how many days was the child physically active (recreational PA) for at least 60 min in the past week? (2) how many days did the child walk or bike to and from school in the past week (active commute) and how many minutes per day were spent on this activity? and (3) how many days did the child participate in physical education (PE) classes in school during the past week and what was the duration of each class in minutes? These questions were adapted from Ecuador’s previous ENSANUT 2012 survey^(36)^. The responses to these three questions were aggregated to estimate total minutes of PA per week (recreational PA minutes/week + PE class minutes/week + active commute minutes/week = total PA minutes/week). We also created an indicator for adherence to PA recommendations by dichotomising total PA minutes/week at 420 min to assess whether children met the Ecuador’s PA recommendations (total minutes of PA ≥ 420 min) or not (total minutes of PA < 420 min) in a week^(16)^.

#### Sedentary behaviour

The daily SB of child participants was obtained by asking the caregiver, in a normal day, how much time (in minutes) the child participant spends seated or resting, watching television, playing video games, talking to friends or other activities that require the child to stay seated including chatting, surfing the internet and sending emails? While WHO currently does not have established thresholds for time spent in SB, spending more than two hours of sedentary time/day has been linked to several negative physical and mental health outcomes in children and youth^(37)^. Accordingly, we categorissed daily sedentary minutes in two groups, ≤120 min and >120 min.

#### Anthropometric indicators of nutritional status

Trained survey anthropometrists measured the standing height (cm) and weight (kg) of child participants using a standardised protocol(33). These measurements were made twice and the average recorded in the database. If the first two measurements differed by ± 0·5 cm (height) or ± 0·5 kg (weight), a third measurement was made and the average value from the two closest values was used. The child weight and height measurements were used to calculate BMI defined as weight (kg)/height (m2). The BMI-for-age z (BAZ) scores and height-for-age z (HAZ) scores were compared with the WHO age- and sex-specific growth reference using the WHO macro for R software(38). Child BMI were classified as underweight (BAZ < –2), normal weight (–2 ≤ BAZ ≤ +1), overweight (BAZ > +1) or obese (BAZ > +2)(39). Children were classified as stunted if their HAZ was < –2(39). Since anthropometric data for 822 participants were missing, the analysis of the anthropometric indicators was based on 22 799 child participants.

#### Covariates

The individual-level characteristics examined for potential inclusion as covariates in the statistical models included child age (5–12 years *v*. 13–17 years), child sex (female *v*. male), any child health problems in the past month (yes *v*. no), mother’s age (years), maternal ethnicity (mestizo ethnic majority group *v*. ethnic minority group, i.e. indigenous or afro-descendants), employment status (full-time housewife *v*. other), marital status (legally married *v*. other) and formal education (primary schooling or less, *v*. secondary or higher schooling). The household-level attributes included urbanicity (urban *v*. rural), region of residence (Pacific Coast, Amazon and Galapagos *v*. Andean Highlands), number of children in the household and household asset index (high *v*. low). The household asset index was created based on ownership of twenty-seven items including cars, motorcycles, household appliances such as refrigerator, stoves and washing machines.^(35)^ In this study, we categorised households in the lowest quartile of the asset index as having a low asset index.

### Data analysis

We used survey-provided sample weights to yield nationally representative estimates. All analyses were conducted in Stata/SE 17·0 (StataCorp LLC) using the *svy* prefix command for survey data analysis. The summary statistics are presented as frequencies with weighted percentages for categorical variables and weighted mean and se for continuous variables. We specified unadjusted and adjusted linear and log-binomial regression models to examine the association of HFI with total minutes of PA/week and adherence to PA recommendations, respectively. Unadjusted and adjusted log-binomial regression models were used to examine the association of HFI with SB and stunting. We conducted unadjusted and adjusted multinomial logistic regression to assess the association of HFI with BMI-for age categories. Results are reported as beta estimates or prevalence ratios (or OR for multinomial logistic) with 95 % CI.

We adjusted for several covariates in separate regression models. In adjusted model 1, we included child age, sex, maternal ethnicity, maternal education and number of children in the household. In adjusted model 2, we included model 1 covariates and added urbanicity and region. In adjusted model 3, in addition to model 1 and 2 covariates, we included household asset index. Considering household asset index may be collinear with HFI, we tested for multicollinearity using variance inflation factor. We found no evidence of multicollinearity in our models with all independent variables having a variance inflation factor value of < 2. Furthermore, we did not have household asset index data for 4372 participants. We conducted a complete case analysis and present results for adjusted model 3 using 19 249 observations. However, we performed a sensitivity analysis by imputing all the missing asset index values as either low or high to estimate the maximum possible difference in results that could be expected based on the exclusion of this missing data. Our sensitivity analysis results are detailed in the Supplementary Materials. Statistical significance for all hypothesis tests was set at *P* < 0·05. All statistical tests were two tailed.

## Results

### Household food insecurity and socio-demographic characteristics

In this study, about one in four child households (24 %) had marginal HFI, while one in five (20 %) had moderate-severe HFI. Nearly half the child participants were female (49 %) and nearly two-thirds (64 %) were between ages 5 and 12 years (Table 1). The table shows that other socio-demographic characteristics differed by HFI status. For example, a greater proportion of children from food-insecure households had mothers who were slightly younger, belonged in an ethnic minority group, were not legally married, had poorer education and were full-time housewives, compared with food-secure counterparts. More children from food-insecure households resided in rural areas, particularly in the Pacific Coast or Amazonian regions of Ecuador and belonged to households having a low asset index, compared with food-secure groups. A higher proportion of food-insecure households reported that their children had experienced some type of health problem during the past 30-day period, and the number of household children was greater in food-insecure households compared with their food-secure counterparts.

**Table 1:** Socio-demographic, general health and physical activity characteristics of Ecuadorian children according to their household food insecurity (HFI) status, (*n* 23 621)*

Characteristics	Full Sample *n* 23 621 *(n* or weighted mean)	Weighted % or SE	Food Secure *n* 12 832 *(n* or weighted mean)	Weighted % or SE	Marginal HFI *n* 5496 *(n* or weighted mean)	Weighted % or SE	Moderate- Severe HFI *n* 5293 *(n* or weighted mean)	Weighted % or SE	*P* value
*Child Sex*									0·26
Male	12 088	51·1	6642	51·5	2772	49·4	2674	51·9	
Female	11 533	48·9	6190	48·5	2724	50·6	2619	48·1	
*Child Age*									0·64
5–12 years	15 424	63·5	8347	63·5	3615	64·2	3462	62·6	
13–17 years	8197	36·5	4485	36·5	1881	35·8	1831	37·4	
*Maternal Age*	34·6	0·1	34·9	0·1	34·3	0·2	34·0	0·2	<0·001
*Maternal ethnicity*									<0·001
Mestizo	19 409	88·2	11 118	90·1	4607	88·7	3684	82·4	
Indigenous/Afro-descendants	4212	11·8	1714	9·9	889	11·3	1609	17·6	
*Maternal education*									<0·001
Primary schooling or less	12 017	50·6	5442	41·3	3079	55·7	3496	64·7	
Secondary schooling or higher	11 604	49·4	7390	58·7	2417	44·3	1797	35·3	
*Maternal marital status*									0·001
Legally married	18 534	77·3	10 283	78·8	4297	77·3	3954	73·2	
Other||	5087	22·7	2549	21·2	1199	22·7	1339	26·8	
*Maternal employment*									<0·001
Full time housewife	14 782	55·0	7825	52·2	3524	56·8	3433	60·9	
Other¶	8839	45·0	5007	47·8	1972	43·2	1860	39·1	
*No. of household kids*	2·5	0·0	2·4	0·0	2·5	0·0	2·7	0·0	<0·001
*Household asset index* †									<0·001
Low	5487	32·0	2310	24·6	1534	36·0	1643	48·4	
High	13 762	68·0	8241	75·4	2934	64·0	2587	51·6	
*Urbanicity*									<0·001
Urban	14 357	69·2	8570	73·8	3139	64·1	2648	62·6	
Rural	9264	30·8	4262	26·2	2357	35·9	2645	37·4	
*Region of residence*									<0·001
Andean highland	8877	44·1	4926	45·1	2189	45·2	1762	39·9	
Pacific Coast	8343	49·9	4439	49·3	2107	49·9	1797	51·6	
Amazon	5589	5·8	2795	5·3	1092	4·8	1702	8·4	
Galapagos Islands	812	0·2	672	0·3	108	0·1	32·0	0·1	
*Any child health problems in past month*									<0·001
Yes	4496	19·8	2178	17·4	1120	20·5	1198	25·7	
No	19 086	80·2	10 629	82·6	4371	79·5	4086	74·3	
*Total PA mins/week*	259·1	2·4	254·4	3·2	264·4	4·3	265·8	3·8	0·03
*Met PA recommendations*									0·06
Yes	4841	19·4	2515	18·5	1175	20·2	1151	21·2	
No	18 789	80·6	10 317	81·5	4321	79·8	4142	78·8	
*Sedentary behaviour*									<0·01
>120 min/d	6493	32·0	3845	33·6	1411	29·9	1237	30·1	
≤120 min/d	17 128	68·0	8987	66·4	4085	70·1	4056	69·9	
*BMI-for-age* ‡									
Normal	13 741	60·0	7194	58·3	3267	60·7	3280	63·7	0·03
Underweight	366	1·7	196	1·7	72	1·6	98	1·8	
Overweight	5288	24·1	2927	24·9	1227	23·8	1134	22·3	
Obese	3150	14·2	1900	15·1	682	13·9	568	12·2	
*Stunting* §									0·02
Yes	2244	8·7	1128	8·0	531	9·2	585	10·2	
No	20 541	91·3	11 224	92·0	4774	90·8	4543	89·8	

* Bivariate tests conducted using *χ* ^2^ tests for categorical variables and adjusted Wald tests for continuous variables.

† Household asset index *N* 19 249.

‡ BMI-for-age *N* 22 545.

§ Stunting *N* 22 785.

|| Other marital status options include single, separated, divorced, widowed and domestic partnership.

¶ Other maternal employment options include self-employed, employers, salaried workers, domestic employees and unpaid workers.

### Physical activity behaviour

The child participants were reported to average 259 min of PA per week. About one in five (19 %) met Ecuador’s PA recommendations for children (Table 1). After adjusting for covariates in models 1–3, HFI was not associated with minutes of PA nor with meeting PA recommendations (Table 2).

**Table 2:** Household food insecurity (HFI) and its association with physical activity levels and sedentary behaviour in Ecuadorian children (*n* 23 621)

Household Food Insecurity Status	Unadjusted Model		Adjusted Model 1†		Adjusted Model 2‡		Adjusted Model 3§,||	
***Weekly physical activity minutes (Beta Coefficients and 95 % Confidence Intervals)***								
Moderate-severe HFI	11·44*	1·98, 20·91	7·76	–1·96, 17·49	7·17	–2·46, 16·82	5·55	–5·01, 16·11
Marginal HFI	9·99*	0·20, 19·78	9·32	–0·41, 19·05	8·48	–1·19, 18·17	5·69	–5·32, 16·71
Food secure	1		1		1		1	
***Weekly adherence to physical activity recommendations (Prevalence Ratio, 95 % Confidence Intervals)***								
Moderate-severe HFI	1·15*	1·02, 1·29	1·11	0·98, 1·25	1·10	0·98, 1·24	1·08	0·95, 1·23
Marginal HFI	1·09	0·96, 1·25	1·08	0·95, 1·23	1·08	0·95, 1·22	1·06	0·92, 1·22
Food secure	1		1		1		1	
***Daily sedentary behaviour (Prevalence Ratio, 95 % Confidence Intervals)***								
Moderate-Severe HFI	0·90*	0·82, 0·98	0·95	0·87, 1·04	0·97	0·89, 1·06	0·99	0·90, 1·10
Marginal HFI	0·89*	0·81, 0·98	0·93	0·84, 1·02	0·95	0·87, 1·04	0·98	0·89, 1·08
Food secure	1		1		1		1	

* *P* < 0·05.

† Adjusted for child age, child sex, maternal ethnicity, maternal education and number of children in the household.

‡ Adjusted for model 1 covariates + urbanicity and region.

§ Adjusted for model 1 and model 2 covariates + household asset index.

|| Adjusted model 3 has *N* 19 249.

### Sedentary behaviour

Approximately one out of every three children (32 %) in the study were reported as being sedentary for 2 hours or more per day (Table 1). After adjustment for covariates, HFI was not associated with SB in the child participants in models 1–3 (Table 2).

### BMI-for-age

In this study, three out of five (60 %) children had normal weight, while 24 % were overweight, 14 % were obese and 1·7 % were underweight (Table 1). After adjusting for covariates in models 1 and 2, moderate-severe HFI was associated with lower prevalence of overweight and obesity in the children (Table 3). However, after including the asset index in the adjustment set in model 3, HFI was no longer associated with overweight and obesity. HFI was not associated with underweight in any of the regression models.

**Table 3:** Household food insecurity (HFI) and its association with anthropometric indicators of nutritional status in Ecuadorian children (*n* 22 799) †

Household Food Insecurity Status	Unadjusted Model		Adjusted Model 1¶		Adjusted Model 2**		Adjusted Model 3*†,||	
***BMI-for-age (Odds Ratio, 95 % Confidence Intervals)*** ‡								
**Underweight**								
Moderate-severe HFI	0·96	0·64, 1·43	0·94	0·61, 1·44	0·95	0·62, 1·46	0·75	0·45, 1·24
Marginal HFI	0·90	0·57, 1·43	0·90	0·55, 1·45	0·92	0·57, 1·50	0·79	0·44, 1·41
Food secure	1		1		1		1	
**Overweight**								
Moderate-Severe HFI	0·82*	0·72, 0·94	0·85*	0·74, 0·98	0·85*	0·74, 0·98	0·90	0·77, 1·05
Marginal HFI	0·92	0·80, 1·06	0·93	0·81, 1·07	0·94	0·81, 1·08	0·95	0·81, 1·11
Food secure	1		1		1		1	
**Obesity**								
Moderate-severe HFI	0·74*	0·61, 0·89	0·81*	0·67, 0·98	0·81*	0·67, 0·98	0·88	0·71, 1·08
Marginal HFI	0·89	0·73, 1·07	0·92	0·75, 1·11	0·93	0·76, 1·13	1·02	0·83, 1·25
Food secure	1		1		1		1	
***Stunting (Prevalence Ratio, 95 % Confidence Intervals)*** §								
Moderate-severe HFI	1·28*	1·09, 1·51	1·13	0·96, 1·34	1·13	0·96, 1·33	1·07	0·88, 1·30
Marginal HFI	1·16	0·96, 1·40	1·09	0·90, 1·33	1·08	0·86, 1·32	1·04	0·82, 1·31
Food secure	1		1		1		1	

* *P* < 0·01.

† Data for 822 child participants were missing.

‡ *n* = 22 545 since 254 participant’s BMI-for-age values were flagged as improbable.

§ *n* = 22 785 since fourteen participant’s height-for-age values were flagged as improbable.

|| For adjusted model 3, *n* (BMI-for-age) = 19 044 and *n* (stunting) = 19 236.

¶ Adjusted for child age, child sex, maternal ethnicity, maternal education and number of children in the household.

** Adjusted for model 1 covariates + urbanicity and region.

*† Adjusted for model 1 and model 2 covariates + household asset index.

### Stunting

Stunting was present in < 10 % of the child participants (Table 1). In our covariate adjusted models 1–3, moderate-severe HFI was not associated with stunting (Table 3).

### Sensitivity analysis

The magnitude and precision of our estimates after imputing missing asset index value as high or low were similar to the adjusted models excluding the missing asset index data (see online supplementary material, Supplementary Analysis Tables 1–4). Children from households with missing asset index data were more likely to present with stunting but had lower total PA minutes per week and were less likely to meet the PA recommendations. HFI, SB and BMI-for-age were not significantly different between those with and without missing asset index data (see online supplementary material, Supplementary Analysis Table 6). Moreover, we found that HFI was not associated with PE class minutes/week nor with recreational PA minutes/week separately (see online supplementary material, Supplementary Analysis Table 5). However, moderate-severe HFI was associated with an average of 7·4 (95 % CI: 3·46, 11·26) more minutes/week spent in active commuting to and from school. In sex-stratified analysis, HFI was not associated with PA, SB, overweight/obesity, nor stunting (see online supplementary material, Supplementary Analysis Tables 7 and 8). However, in males, moderate-severe HFI was associated with 0·51 times the odds of underweight, while marginal HFI was associated with 0·47 times the odds of underweight in females (see online supplementary material, Supplementary Analysis Table 8) after adjusting for asset index.

## Discussion

This study identified a high prevalence of HFI in the households of 5–17-year-old child participants. The high HFI prevalence is consistent with that reported by previous surveys and other studies for Ecuadorian households^(19,22–24)^. The major findings of this study were that HFI was not associated with reported PA, SB, stunting or overweight and obesity in the Ecuadorian child and adolescents surveyed in the 2018 ENSANUT. These findings suggest that while HFI continues to be a burden in Ecuadorian households, it does not appear to contribute towards the physical inactivity and SB reported for this group of Ecuadorian children and adolescents.

The null associations identified in our study between HFI with total minutes of PA and meeting PA recommendations differ from our *apriori* hypothesis. However, they are consistent with the evidence published on HFI and PA in Brazilian^(11)^, U.S.^(10)^ and Spanish^(40)^ children and adolescents. They also partially concur with a U.S. study reporting that food-insecure children did not differ from their food-secure counterparts regarding meeting physical activity guidelines although they engaged in fewer minutes of moderate to vigorous physical activity^(6)^. Another study also found that although HFI was not associated with physical activity, it was associated with attending more PE classes and with higher odds of active commuting to school^(12)^.

In our post hoc analyses, we found that children from moderate-severe HFI households had higher active commuting minutes than those from food-secure households. It is plausible that children from resource constrained, food-insecure households might not have had extra money for public transport or access to carpools and other forms of passive transport. Therefore, they accumulated more active commuting minutes by walking or biking to school, which is a low-cost alternative^(12)^. However, it is important to note that our findings imply that, on average, children from food-insecure households actively commuted about one extra minute per day compared with children from food-secure households, which may not be a meaningful difference.

Different from our findings, other studies have reported negative associations between HFI and physical activity^(6,9,13)^. The inconsistent findings they reported for this relationship could be due to the different methods used to define and measure physical activity. For instance, some studies focused on recreational physical activity^(8)^, one included active commute time and time spent in PE classes^(10)^, while another relied on objective measurements obtained from accelerometers^(6)^. Differing methods were also used by the various studies to measure food insecurity. Some relied on a single question that asked how often adolescents had been hungry during the past 30 d^(12,13)^, while others used either the full version of the US Department of Agriculture’s eighteen-item Household Food Security Survey Module^(6)^ or an abbreviated^(8,10)^, or an adapted version^(11)^ of the same. Furthermore, two studies measured individual-level child food insecurity instead of HFI^(7,9)^. In addition, differences in geographical location, environmental and infrastructural factors, attitudes towards physical activity behaviour and access to TV and video games among the study populations between HIC and LMIC may be responsible for the mixed findings for this relationship^(41)^.

Also different from what we hypothesised, HFI was not associated with child SB. Our results are similar to previous findings from Brazil^(11)^ and the USA^(6,10)^ reporting that children from food-insecure households were not more likely to be sedentary than their food-secure counterparts. One study used school-based data from Global Student Health Surveys conducted in sixty-six LMIC. Their findings indicated that food insecurity in adolescents living in upper-middle income and low-income countries was not associated with SB.^(41)^ This contrasts with the results from other studies suggesting that HFI was associated with higher^(40)^ or lower^(12)^ SB. Since more than two-thirds (68 %) of our study participants had less than 2 hours of daily SB, it is possible that the Ecuadorian population is at an earlier stage of epidemiologic transition characterised by a lower prevalence of SB among children and adolescents. Furthermore, Ecuadorian adolescents may have to assist with household chores, cooking and particularly in rural areas, help with farming, which reduces the time available for sedentary activities^(42)^.

The evidence of an association between HFI and overweight/obesity in this age group is ambiguous. However, studies suggest that adolescent girls from food-insecure households may be at risk of overweight/obesity compared with their food-secure counterparts^(43,44)^. This relationship could be further complicated by the Ecuador’s economic development level^(44)^. Most studies that have found a positive association of HFI with overweight/obesity were situated in high-income country settings^(40,44,45)^. Fewer studies from LMIC have found a similar positive relationship,^(46)^ and some have reported negative associations^(44,45)^. This inconsistency could be due to different pathways that link HFI, socio-economic status, the stage of nutritional transition and overweight/obesity in children from HIC *v*. LMIC. One study investigating this relationship in Quebec, Canada (HIC setting) and Jamaica (LMIC setting) found that HFI raised the risk of overweight/obesity in children in the former, but was negatively associated with overweight/obesity in the latter^(45)^. The current study findings, while contrary to our hypothesis, tend to align with the published evidence. We found HFI was associated with lower prevalence of overweight and obesity in children and adolescents. However, adjustment for household asset index attenuated the findings, and HFI was no longer associated with overweight/obesity. It is possible that it may have confounded the relationship between HFI and overweight/obesity in the other regression models. However, it is also crucial to consider that the asset index, a proxy for socio-economic status, may explain some of the effect of HFI on overweight/obesity in our study. It seems likely that the food-insecure households of Ecuadorian children may not have the economic resources to purchase more obesogenic processed and ultra-processed food and beverages which can be more expensive than traditional diet in this setting. It has been reported that in Colombia, another upper middle-income Andean country, children from food-insecure households consumed fewer snacks and processed food than children from food-secure households^(47)^. This contrasts with evidence from HIC where those from food-insecure households have easier availability and accessibility to fast food, which can promote weight gain^(48)^. Furthermore, our child participants may have actively commuted to school due to it being a cheaper or only option than passive modes of transportation which slightly increased their physical activity. This may have contributed to the low prevalence of overweight/obesity observed in Ecuadorian child participants living in from households with marginal and moderate-severe food-insecure households compared with their food-secure counterparts. Interestingly, after adjusting for asset index, both males and females from food-insecure households had lower odds of underweight than those from food-secure households. However, these results should be interpreted with caution due to the low prevalence of underweight reported in this study, which may have biased our results.

The findings from some prior studies suggest that HFI appears to be positively associated with stunting in children. However, this direction of association was predominantly reported in LMIC rather than HIC^(44,49)^. Differences in the absolute severity of HFI, different coping strategies and local safety net programs may partially explain these mixed findings^(50)^. Our findings indicate that HFI was not associated with stunting in our child participants. While contrary to our hypothesis, our results are consistent with previous literature from Latin America that have also reported null findings between food insecurity and child stunting^(22,47)^. While stunting continues to be a burden in Ecuador, its aetiology is multifactorial and there may be other determinants such as access to water and safe sanitation that was not assessed in this study. Furthermore, stunting in LMIC settings is often the consequence of chronic undernutrition and repeated infections. Thus, this cross-sectional study may not have been able to capture the association between HFI and stunting.

## Strengths and limitations

Our study has several strengths. One of these is that we used data from a nationally representative survey. The ENSANUT-2018 had a low non-response rate (1·2 %), achieved high national coverage (∼92 %) and used standardised data collection procedures and quality control methods to ensure data quality^(33)^. The child risk module used for the analysis had a large sample size (*n* 23 621), which permitted the adequate testing of our study hypotheses. In addition, physical activity was more broadly defined than some previous studies, including recreational physical activity, PE classes and active commuting, which is more closely aligned to the WHO definition. The survey used the FIES, an experience-based food security survey module to measure HFI. This allowed for us to identify children from households with both marginal and moderate-severe HFI. Also, height and weight were objectively measured by trained survey anthropometrists rather than relying on self-reported measurements which are not as accurate. Finally, this study extends prior work by assessing the relationship of HFI with physical activity and SB, which, to our knowledge, has not yet been studied in Ecuadorian children.

However, our study also has several limitations. One of the study limitations was that caregiver-reported data were used to assess HFI, physical activity and SB. Caregivers could have over- or under-reported their household’s food insecurity as well as over- or under-estimated the physical activity and SB of their children. These types of self-reports could have been affected by social desirability and recall bias. Also, the caregivers reported food insecurity at the household level which might not accurately reflect the individual food security situation of the child and adolescent participants. Another limitation is that the ENSANUT-2018 measured HFI for the previous 12-month period. Therefore, it might not reflect the HFI situation over a longer period which may be a concern since anthropometric indicators of nutritional status such as stunting, overweight and obesity can develop over a long time. Our study did not identify associations between HFI and underweight which may develop over a longer period of time. Considering that < 2 % of our sample were reported as underweight, it is possible that this study was not adequately powered to detect differences in child underweight across HFI groups.

Moreover, unmeasured confounders could have affected the results. Our analyses were restricted to variables contained in the database. Although the ENSANUT-2018 survey collected limited data on the intakes of a few types of foods, it did not collect data on the dietary protein and micronutrient (e.g. Fe, Zn and vitamin A) intakes of the child and adolescent participants. Diet plays a significant role in not only BMI-for-age and stunting but also on physical activity and SB. While in this study, we hypothesise that diet likely mediates some of the pathway between HFI and our study outcomes, future research should incorporate dietary data in their analysis of HFI and physical activity and SB to get a more in-depth understanding of the mechanisms for this relationship. Finally, the cross-sectional nature of this study allows us to infer but not establish causal effects.

### Conclusion

Our study findings suggest HFI is not associated with physical activity, SB, stunting nor overweight/obesity in Ecuadorian children and adolescents. However, a notable prevalence of insufficient physical activity was reported among Ecuadorian children and adolescents aged 5–17 years. Thus, it is imperative to enhance the monitoring of physical activity levels and comprehensively examine the underlying factors that impede or facilitate physical activity in this particular age group. Leveraging this evidence can inform the design of interventions and evidence-based policies aimed at fostering physical activity and reducing SB, which can promote the health and well-being of Ecuadorian children and adolescents.

## Supporting information

Chakraborty et al. supplementary material Chakraborty et al. supplementary material

## Supplementary material

For supplementary material accompanying this paper visit https://doi.org/10.1017/S1368980024000351

## References

[1] Janssen I & LeBlanc AG (2010) Systematic review of the health benefits of physical activity and fitness in school-aged children and youth. Int journal behavioral nutrition physical activity 7, 1–16. 10.1186/1479-5868-7-40 PMC2885312 20459784

[2] WHO (2010) Global Recommendations on Physical Activity for Health. Geneva: World Heal Organization. 26180873

[3] Guthold R , Stevens GA , Riley LM et al. (2020) Global trends in insufficient physical activity among adolescents: a pooled analysis of 298 population-based surveys with 1 6 million participants. Lancet Child Adolesc Health 4, 23–35. 31761562 10.1016/S2352-4642(19)30323-2 PMC6919336

[4] Aguilar-Farias N , Martino-Fuentealba P , Carcamo-Oyarzun J et al. (2018) A regional vision of physical activity, sedentary behaviour and physical education in adolescents from Latin America and the Caribbean: results from 26 countries. Int J Epidemiol 47, 976–986. 29554308 10.1093/ije/dyy033

[5] Bickel G , Mark N , Cristofer P , William H & John C (2000) Guide to Measuring Household Food Security, Revised March 2000; available at https://fns-prod.azureedge.us/sites/default/files/FSGuide.pdf (accessed January 2023).

[6] To QG , Frongillo EA , Gallegos D et al. (2014) Household food insecurity is associated with less physical activity among children and adults in the U.S. population. J Nutr 144, 1797–1802. 25332479 10.3945/jn.114.198184

[7] Shanafelt A , Hearst MO , Wang Q et al. (2016) Food insecurity and rural adolescent personal health, home, and academic environments. J school na 86, 472–480. 10.1111/josh.12397 PMC4852387 27122147

[8] Gulliford MC , Nunes C & Rocke B (2006) Food insecurity, weight control practices and body mass index in adolescents. Public Health Nutr 9, 570–574. 16923288 10.1079/phn2005886

[9] Fram MS , Ritchie LD , Rosen N et al. (2015) Child experience of food insecurity is associated with child diet and physical activity. J Nutr 145, 499–504. 25733465 10.3945/jn.114.194365

[10] Navarro SM , Tsai MM , Ritchie LD et al. (2022) Household food insecurity and children’s physical activity and sedentary behavior in the United States: the healthy communities study. Public Health Nutr 25, 381–388. 34108064 10.1017/S1368980021002536 PMC8660938

[11] Lopes TS , Sichieri R , Salles-Costa R et al. (2013) Family food insecurity and nutritional risk in adolescents from a low-income area of Rio de Janeiro, Brazil. J Biosocial Sci 45, 661–674. 10.1017/S0021932012000685 23149069

[12] Araujo RHO , Werneck AO , Barboza LL et al. (2022) Prevalence and sociodemographic correlates of physical activity and sitting time among South American adolescents: a harmonized analysis of nationally representative cross-sectional surveys. Int J Behav Nutr Phys Act 19, 52. 35527268 10.1186/s12966-022-01291-3 PMC9080195

[13] Fram MS , Nguyen HT & Frongillo EA (2022) Food insecurity among adolescent students from 95 countries is associated with diet, behavior, and health, and associations differ by student age and sex. Curr Dev Nutr 6, nzac024. 35317415 10.1093/cdn/nzac024 PMC8929982

[14] Freire WB , Silva-Jaramillo KM , Ramírez-Luzuriaga MJ et al. (2014) The double burden of undernutrition and excess body weight in Ecuador. Am J Clin Nutr 100, 1636s–1643s. 25411306 10.3945/ajcn.114.083766

[15] Abril V , Manuel-y-keenoy B , Solà R et al. (2013) Prevalence of overweight and obesity among 6-to 9-year-old school children in Cuenca, Ecuador: relationship with physical activity, poverty, and eating habits. Food Nutr Bull 34, 388–401. 24605689 10.1177/156482651303400404

[16] Ministerio de Salud Pública & Ministerio de Educación (2017) Guía de alimentación para padres de familia. Quito, Ecuador: Ministerio de Salud Pública and Ministerio de Educación.

[17] Andrade S , Ochoa-Avilés A , Freire W et al. (2018) Results from Ecuador’s 2018 report card on physical activity for children and youth. J Phys Act Health 15, S344–S346. 30475106 10.1123/jpah.2018-0536

[18] Thompson AL , Nicholas KM , Watson E et al. (2020) Water, food, and the dual burden of disease in Galápagos, Ecuador. Am J Hum Biol 32, e23344. 31642150 10.1002/ajhb.23344 PMC7114884

[19] FAO, IFAD, UNICEF, WFP & WHO (2022) The State of Food Security and Nutrition in the World 2022. Repurposing Food and Agricultural Policies to make Healthy Diets more Affordable. Rome: FAO.

[20] Weigel MM & Armijos RX (2018) Household food insecurity and psychosocial dysfunction in ecuadorian elementary schoolchildren. Int J Pediatr 2018, 6067283. 30186331 10.1155/2018/6067283 PMC6110046

[21] Weigel MM & Armijos RX (2023) Food insecurity is associated with self-reported oral health in school-age Ecuadorian children and is mediated by dietary and non-dietary factors. Public Health Nutr 26, 23–32. 36172927 10.1017/S1368980022002166 PMC11077448

[22] Walrod J , Seccareccia E , Sarmiento I et al. (2018) Community factors associated with stunting, overweight and food insecurity: a community-based mixed-method study in four Andean indigenous communities in Ecuador. BMJ Open 8, e020760. 10.1136/bmjopen-2017-020760 PMC6042540 29982205

[23] Weigel MM & Armijos MM (2015) Food insufficiency in the households of reproductive-age Ecuadorian women: association with food and nutritional status indicators. Ecol Food Nutr 54, 20–42. 25347579 10.1080/03670244.2014.953249

[24] Velez Pinos P & Buenano Rodriguez C (2019) Estado de seguridad alimentaria en niños hospitalizados en pediatria y su asociación con malnutrición. Azogues-Ecuador, 2017. Rev. Ecuat. Pediatr 20, 14–19.

[25] Bergmans RS , Palta M , Robert SA et al. (2018) Associations between food security status and dietary inflammatory potential within lower-income adults from the united states national health and nutrition examination survey, cycles 2007 to 2014. J Acad Nutr Diet 118, 994–1005. 29452975 10.1016/j.jand.2017.12.003 PMC5971121

[26] Eicher-Miller HA , Mason AC , Weaver CM et al. (2009) Food insecurity is associated with iron deficiency anemia in US adolescents. Am J Clin Nutr 90, 1358–1371. 19776137 10.3945/ajcn.2009.27886

[27] Wang Y , Armijos RX & Weigel M-M (2023) Dietary inflammatory index and cardiometabolic risk in ecuadorian school-age children. J Am Nutr Assoc 42, 618–627. 35980812 10.1080/27697061.2022.2113177

[28] Malina RM & Katzmarzyk PT (2006) Physical activity and fitness in an international growth standard for preadolescent and adolescent children. Food Nutr Bull 27, S295–313. 17361664 10.1177/15648265060274S511

[29] Fram MS , Frongillo EA , Jones SJ et al. (2011) Children are aware of food insecurity and take responsibility for managing food resources. J Nutr 141, 1114–1119. 21525257 10.3945/jn.110.135988

[30] Laraia BA (2013) Food insecurity and chronic disease. Adv Nutr 4, 203–212. 23493536 10.3945/an.112.003277 PMC3649100

[31] Lycett KM , Wijayawickrama DJ , Liu M et al. (2022) Does an inflammatory diet affect mental well-being in late childhood and mid-life? A cross-sectional study. Br J Nutr 127, 939–947. 33998415 10.1017/S0007114521001616

[32] Bernal J , Frongillo EA , Herrera HA et al. (2014) Food insecurity in children but not in their mothers is associated with altered activities, school absenteeism, and stunting. J Nutr 144, 1619–1626. 25143373 10.3945/jn.113.189985

[33] INEC (2018) Ecuador - ENSANUT; available at https://www.ecuadorencifras.gob.ec/salud-salud-reproductiva-y-nutricion/ (accessed September 2022).

[34] FAO (2022) Escala De Inseguridad Alimentaria Basada En La Experiencia; available at https://www.fao.org/3/bl404s/bl404s.pdf (accessed December 2022).

[35] Weigel MM & Armijos RX (2022) The ecuadorian school food environment: association with healthy and unhealthy food and beverage consumption and BMI. Food Nutr Bull 43, 439–464. 35993259 10.1177/03795721221116447

[36] INEC (2023) Evolución histórica de la Encuesta Nacional de Salud y Nutrición 2018. Available at https://www.ecuadorencifras.gob.ec/documentos/web-inec/Estadisticas_Sociales/ENSANUT/ENSANUT_2018/Evolucion%20Historica%20de%20ENSANUT%202018.pdf (accessed December 2023).

[37] Tremblay MS , LeBlanc AG , Kho ME et al. (2011) Systematic review of sedentary behaviour and health indicators in school-aged children and youth. Int J Behav Nutr Physical Activity 8, 98. 10.1186/1479-5868-8-98 PMC3186735 21936895

[38] Canadian Pediatric Endocrine Group (2007) Calculator: WHO 2007 Z-scores; available at https://cpeg-gcep.shinyapps.io/who2007_cpeg/ (accessed November 2022).

[39] de Onis M , Onyango AW , Borghi E et al. (2007) Development of a WHO growth reference for school-aged children and adolescents. Bull World Health Organ 85, 660–667. 18026621 10.2471/BLT.07.043497 PMC2636412

[40] Ortiz-Marrón H , Ortiz-Pinto MA , Urtasun Lanza M et al. (2022) Household food insecurity and its association with overweight and obesity in children aged 2 to 14 years. BMC Public Health 22, 1930. 36253730 10.1186/s12889-022-14308-0 PMC9578200

[41] Vancampfort D , Van Damme T , Firth J et al. (2019) Correlates of leisure-time sedentary behavior among 181,793 adolescents aged 12–15 years from 66 low- and middle-income countries. PloS one 14, e0224339. 31725744 10.1371/journal.pone.0224339 PMC6855478

[42] Van Royen K , Verstraeten R , Andrade S et al. (2015) Factors affecting physical activity in ecuadorian adolescents: a focus group study. J Physical Activity Health 12, 340–348. 10.1123/jpah.2013-0288 24956609

[43] Frongillo EA & Bernal J (2014) Understanding the coexistence of food insecurity and obesity. Curr Pediatr Rep 2, 284–290.

[44] Maitra C (2018) *A Review of Studies Examining the Link Between Food Insecurity and Malnutrition*. Rome: Technical Paper FAO.

[45] Dubois L , Francis D , Burnier D et al. (2011) Household food insecurity and childhood overweight in Jamaica and Québec: a gender-based analysis. BMC Public Health 11, 199. 21453491 10.1186/1471-2458-11-199 PMC3078098

[46] Schlüssel MM , Silva AA , Pérez-Escamilla R et al. (2013) Household food insecurity and excess weight/obesity among Brazilian women and children: a life-course approach. Cad Saude Publica 29, 219–226. 23459802 10.1590/s0102-311x2013000200003

[47] Isanaka S , Mora-Plazas M , Lopez-Arana S et al. (2007) Food insecurity is highly prevalent and predicts underweight but not overweight in adults and school children from Bogotá, Colombia. J Nutr 137, 2747–2755. 18029494 10.1093/jn/137.12.2747

[48] Ma X , Liese AD , Bell BA et al. (2016) Perceived and geographic food access and food security status among households with children. Public Health Nutr 19, 2781–2788. 27133939 10.1017/S1368980016000859 PMC5588026

[49] Moradi S , Mirzababaei A , Mohammadi H et al. (2019) Food insecurity and the risk of undernutrition complications among children and adolescents: a systematic review and meta-analysis. Nutr 62, 52–60. 10.1016/j.nut.2018.11.029 30852458

[50] Hadley C & Crooks DL (2012) Coping and the biosocial consequences of food insecurity in the 21st century. Am J Phys Anthropol 149, 72–94. 23109261 10.1002/ajpa.22161

