# Supplementary material for: Household food insecurity and physical activity behaviour in Ecuadorian children and adolescents: findings from the Ecuador 2018 National Health and Nutrition Survey (ENSANUT-2018)
Source: Public Health Nutr. 2024 Feb 2;27(1):e67. doi: 10.1017/S1368980024000351 (PMC10966927; doi:10.1017/S1368980024000351)
Supplement: Chakraborty et al. supplementary material [file S1368980024000351sup001.docx]

**Supplementary Analyses**

Results are reported as beta estimates or prevalence ratios (or odds ratios for multinomial logistic) with 95% confidence intervals.

Beta estimates show by how much, on average, the response variable changes with one-unit change in the predictor variable, while prevalence and odds ratios are measures of association that compare outcome occurrence in food secure, marginal HFI, and moderate-severe HFI groups.

Table 1: HFI and its association with physical activity levels and sedentary behavior in Ecuadorian children with missing asset index data imputed as low (N = 23,621)

| **Household Food Insecurity Status** | **Adjusted Model 3^3^** |
| --- | --- |
| ***Weekly physical activity minutes (beta coefficients and 95% confidence intervals)*** | |
| Moderate-Severe HFI | 8.589 (-0.98, 18.14) |
| Marginal HFI | 8.97 (-0.75, 18.7) |
| Food Secure | 1 |
| ***Weekly adherence to physical activity recommendations (Prevalence Ratio, 95% confidence intervals)*** | |
| Moderate-Severe HFI | 1.10 (0.97, 1.24) |
| Marginal HFI | 1.07 (0.95, 1.22) |
| Food Secure | 1 |
| ***Daily sedentary behavior (Prevalence Ratio, 95% confidence intervals)*** | |
| Moderate-Severe HFI | 1.00 (0.92, 1.10) |
| Marginal HFI | 0.96 (0.88, 1.05) |
| Food Secure | 1 |

^3^adjusted for child age, child sex, maternal ethnicity, maternal education, and number of children in the household, urbanicity, region, and asset index

Table 2: HFI and its association with physical activity levels and sedentary behavior in Ecuadorian children with missing asset index data imputed as high (N = 23,621)

| **Household Food Insecurity Status** | **Adjusted Model 3^3^** |
| --- | --- |
| ***Weekly physical activity minutes (beta coefficients and 95% confidence intervals)*** | |
| Moderate-Severe HFI | 2.26 (-7.39, 22.89) |
| Marginal HFI | 6.41 (-3.18, 15.99) |
| Food Secure | 1 |
| ***Weekly adherence to physical activity recommendations (Prevalence Ratio, 95% confidence intervals)*** | |
| Moderate-Severe HFI | 1.07 (0.95, 1.20) |
| Marginal HFI | 1.06 (0.94, 1.21) |
| Food Secure | 1 |
| ***Daily sedentary behavior (Prevalence Ratio, 95% confidence intervals)*** | |
| Moderate-Severe HFI | 1.00 (0.92, 1.10) |
| Marginal HFI | 0.96 (0.88, 1.05) |
| Food Secure | 1 |

^3^adjusted for child age, child sex, maternal ethnicity, maternal education, and number of children in the household, urbanicity, region, and asset index

Table 3: Household food insecurity (HFI) and its association with anthropometric indicators of nutritional status in Ecuadorian children with missing asset index data imputed as low, (N = 22,799)

| **Household Food Insecurity Status** | **Adjusted Model 3^3, c^** |
| --- | --- |
| ***BMI- for-age (Prevalence Ratio, 95% confidence intervals) ^a^*** | |
| **Underweight** | |
| Moderate-Severe HFI | 0.87 (0.59, 1.38) |
| Marginal HFI | 0.87 (0.56, 1.46) |
| Food Secure | 1 |
| **Overweight** | |
| Moderate-Severe HFI | 0.86 (0.75, 0.99) * |
| Marginal HFI | 0.94 (0.81, 1.08) |
| Food Secure | 1 |
| **Obese** | |
| Moderate-Severe HFI | 0.81 (0.67, 0.99) * |
| Marginal HFI | 0.93 (0.76, 1.13) |
| Food Secure | 1 |
| ***Stunting (Prevalence Ratio, 95% confidence intervals) ^b^*** | |
| Moderate-Severe HFI | 1.07 (0.91, 1.27) |
| Marginal HFI | 1.06 (0.87, 1.29) |
| Food Secure | 1 |

^3^adjusted for child age, child sex, maternal ethnicity, maternal education, and number of children in the household, urbanicity, region, and asset index

^a^N = 22545 since 254 participant’s BMI-for-age values were flagged as improbable

^b^N = 22785 since 14 participant’s height-for-age values were flagged as improbable

^c^For adjusted Model 3, N (BMI-for-age) = 19044 and N (Stunting) = 19236

*p< 0.05

Table 4: Household food insecurity (HFI) and its association with anthropometric indicators of nutritional status in Ecuadorian children with missing asset index data imputed as high, (N = 22,799)

| **Household Food Insecurity Status** | **Adjusted Model 3^3^** |
| --- | --- |
| ***BMI- for-age (Prevalence Ratio, 95% confidence intervals) ^a^*** | |
| **Underweight** | |
| Moderate-Severe HFI | 0.89 (0.59, 1.33) |
| Marginal HFI | 0.87 (0.56, 1.36) |
| Food Secure | 1 |
| **Overweight** | |
| Moderate-Severe HFI | 0.86 (0.75, 0.99)* |
| Marginal HFI | 0.94 (0.81, 1.08) |
| Food Secure | 1 |
| **Obese** | |
| Moderate-Severe HFI | 0.85 (0.70, 1.02) |
| Marginal HFI | 0.92 (0.76, 1.11) |
| Food Secure | 1 |
| ***Stunting (Prevalence Ratio, 95% confidence intervals) ^b^*** | |
| Moderate-Severe HFI | 1.10 (0.93, 1.30) |
| Marginal HFI | 1.07 (0.87, 1.30) |
| Food Secure | 1 |

^3^adjusted for child age, child sex, maternal ethnicity, maternal education, and number of children in the household, urbanicity, region, and asset index

^a^N = 22545 since 254 participant’s BMI-for-age values were flagged as improbable

^b^N = 22785 since 14 participant’s height-for-age values were flagged as improbable

^c^For adjusted Model 3, N (BMI-for-age) = 19044 and N (Stunting) = 19236

Table 5: Household food insecurity (HFI) and its association with recreational physical activity, active commute, and physical education classes in Ecuadorian children (N = 23,621)

| **Household Food Insecurity Status** | **Unadjusted Model** | **Adjusted Model 1^1^** | **Adjusted Model 2^2^** | **Adjusted Model 3^3,#^** |
| --- | --- | --- | --- | --- |
| ***Recreational physical activity minutes/week (beta coefficients and 95% confidence intervals)*** | | | | |
| Moderate-Severe HFI | 4.64 (-3.45, 12.72) | 2.24 (-6.12, 10.60) | 1.53 (-6.73, 9.79) | -0.42 (-9.69, 8.86) |
| Marginal HFI | 8.19 (-0.66, 17.05) | 8.04 (-0.84, 16.92) | 7.16 (-1.66, 15.98) | 6.68 (-3.52, 16.87) |
| Food Secure | 1 | 1 | 1 | 1 |
| ***Active commute minutes/week (beta coefficients and 95% confidence intervals)*** | | | | |
| Moderate-Severe HFI | 11.39 (8.12, 14.65) ** | 8.49 (5.15, 11.83) ** | 8.11 (4.76, 11.48) ** | 7.36 (3.46, 11.26) ** |
| Marginal HFI | 3.09 (0.19, 5.97) * | 1.77 (-1.08,4.61) | 1.52 (-1.30, 4.35) | -0.42 (-3.42, 2.57) |
| Food Secure | 1 | 1 | 1 | 1 |
| ***School physical education classes minutes/week (beta coefficients and 95% confidence intervals)*** | | | | |
| Moderate-Severe HFI | -4.58 (-7.10, -2.06) ** | -2.97 (-5.53, -0.40) * | -2.47 (-4.99, 0.05) | -1.39 (-3.92, 1.14) |
| Marginal HFI | -1.29 (-3.73, 1.16) | -0.49 (-2.90, 1.92) | -0.20 (-2.61, 2.20) | -0.56 (-3.03, 1.91) |
| Food Secure | 1 | 1 | 1 | 1 |

^1^adjusted for child age, child sex, maternal ethnicity, maternal education, and number of children in the household

^2^adjusted for Model 1 covariates + urbanicity and region

^3^adjusted for Model 1 and Model 2 covariates + household asset index

^#^Adjusted model 3 has N = 19,249

*p<0.05, ** p < 0.001

Table 6: Sociodemographic characteristics based on missing and non-missing asset index data

| **Characteristics** | **Non-Missing**  **N = 19249** | **Missing**  **N = 4372** | **p value** |
| --- | --- | --- | --- |

|  |  |  |
| --- | --- | --- |
|  | ***N (weighted %) or weighted Mean ± SE*** |  |

| *Child Sex* |  |  | 0.91 |
| --- | --- | --- | --- |
| Male | 9880 (51.1) | 2208 (50.9) |  |
| Female | 9369 (48.9) | 2164 (49.1) |  |
| *Child Age* |  |  | 0.07 |
| 5 to 12 years | 12632 (64.0) | 2792 (61.3) |  |
| 13 to 17 years | 6617 (36.0) | 1580 (38.7) |  |
| *Maternal Age* | 34.76 ± 0.09 | 33.64 ± 0.21 | <0.001 |
| *Maternal Ethnicity* |  |  | 0.09 |
| Mestizo | 15899 (88.5) | 3510 (87.0) |  |
| Indigenous/Afro-descendants | 3350 (11.5) | 862 (13.0) |  |
| *Maternal education* |  |  | 0.05 |
| Primary schooling or less | 9656 (48.9) | 2361 (52.1) |  |
| Secondary schooling or higher | 9593 (51.1) | 2011 (47.9) |  |
| *Maternal Marital Status* |  |  | 0.40 |
| Married | 15104 (77.1) | 3430 (78.2) |  |
| Other^1^ | 4145 (22.9) | 942 (21.8) |  |
| *Maternal employment* |  |  | 0.001 |
| Full time housewife | 11970 (54.2) | 2812 (59.1) |  |
| Other^2^ | 7279 (45.8) | 1560 (40.9) |  |
| *No. of household kids* | 2.50 ± 0.01 | 2.49 ± 0.03 | 0.80 |
| *Urbanicity* |  |  | 0.03 |
| Urban | 11823 (69.8) | 2534 (66.7) |  |
| Rural | 7426 (30.2) | 1838 (33.3) |  |
| *Region* |  |  | 0.18 |
| Andean highland | 7165 (43.7) | 1712 (45.9) |  |
| Pacific Coast | 6816 (50.4) | 1527 (47.9) |  |
| Amazon | 4558 (5.7) | 1031 (6.0) |  |
| Galapagos Islands | 710 (0.2) | 102 (0.1) |  |
| *Any child health problems in past month* |  |  | 0.09 |
| Yes | 3693 (20.1) | 803 (18.2) |  |
| No | 15556 (79.9) | 3530 (81.8) |  |
| *Household Food Insecurity* |  |  | 0.37 |
| Food Secure | 10551 (56.0) | 2281 (55.4) |  |
| Marginal HFI | 4468 (24.6) | 1028 (23.5) |  |
| Moderate-Severe HFI | 4230 (19.4) | 1063 (21.1) |  |
| *Total PA mins/week* | 268.49 ± 2.56 | 215.72 ± 4.86 | <0.001 |
| *Met PA recommendations* |  |  | <0.001 |
| Yes | 4064 (20.2) | 777 (15.9) |  |
| No | 15185 (79.8) | 3595 (84.1) |  |
| *Sedentary Behavior* |  |  | 0.12 |
| > 120 minutes/day | 5303 (32.4) | 1190 (30.2) |  |
| ≤ 120 minutes/day | 13946 (67.6) | 3182 (69.8) |  |
| *BMI-for-age ^a^* |  |  |  |
| Normal | 11642 (59.8) | 2099 (56.9) | 0.09 |
| Underweight | 345 (1.9) | 79 (2.2) |  |
| Overweight | 4458 (23.9) | 830 (23.9) |  |
| Obese | 2804 (14.5) | 542 (17.0) |  |
| *Stunting ^b^* |  |  | <0.001 |
| Yes | 1821 (8.4) | 437 (11.0) |  |
| No | 17428 (91.6) | 3113 (89.0) |  |

^a^ BMI-for-age N = 22545

^b^ Stunting N = 22785

^1^Other marital status options include single, separated, divorced, widowed, and domestic partnership.

^2^Other maternal employment options include self-employed and employers/ salaried and domestic employees, and unpaid workers.

Table 7: HFI and its association with physical activity levels and sedentary behavior in Ecuadorian children based on sex (N = 23,621)

| **Household Food Insecurity Status** | **Males**  **Adjusted Model 3** | **Females**  **Adjusted Model 3** |
| --- | --- | --- |
| ***Weekly physical activity minutes (beta coefficients and 95% confidence intervals)*** | | |
| Moderate-Severe HFI | 13.53 (-2.37, 29.45) | -3.21 (-17.3, 10.8) |
| Marginal HFI | 6.51 (-7.29, 20.32) | 4.39 (-10.9, 19.7) |
| Food Secure | 1 | 1 |
| ***Weekly adherence to physical activity recommendations (Prevalence Ratio, 95% confidence intervals)*** | | |
| Moderate-Severe HFI | 1.16 (0.98, 1.36) | 0.96 (0.79, 1.16) |
| Marginal HFI | 1.06 (0.91, 1.24) | 1.06 (0.92, 1.32) |
| Food Secure | 1 | 1 |
| ***Daily sedentary behavior (Prevalence Ratio, 95% confidence intervals)*** | | |
| Moderate-Severe HFI | 0.94 (0.80, 1.07) | 1.06 (0.91, 1.22) |
| Marginal HFI | 0.97 (0.85, 1.10) | 0.99 (0.97, 1.13) |
| Food Secure | 1 | 1 |

Adjusted for: child age, maternal ethnicity, maternal education, and number of children in the household, urbanicity, region, and asset index

Table 8: Household food insecurity (HFI) and its association with anthropometric indicators of nutritional status in Ecuadorian children based on sex, (N = 22,799)

| **Household Food Insecurity Status** | **Males**  **Adjusted Model 3** | **Females**  **Adjusted Model 3** |
| --- | --- | --- |
| ***BMI- for-age (Odds Ratio, 95% confidence intervals)*** | | |
| **Underweight** | | |
| Moderate-Severe HFI | 0.51 (0.28, 0.94)* | 1.16 (0.57, 2.36) |
| Marginal HFI | 1.07 (0.54, 2.11) | 0.47 (0.23, 0.95)* |
| Food Secure | 1 | 1 |
| **Overweight** | | |
| Moderate-Severe HFI | 0.96 (0.76, 1.21) | 0.85 (0.67, 1.06) |
| Marginal HFI | 1.11 (0.89, 1.38) | 0.82 (0.66, 1.02) |
| Food Secure | 1 | 1 |
| **Obesity** | | |
| Moderate-Severe HFI | 1.11 (0.84, 1.47) | 0.87 (0.63, 1.29) |
| Marginal HFI | 0.89 (0.68, 1.18) | 0.87 (0.66, 1.15) |
| Food Secure | 1 | 1 |
| ***Stunting (Prevalence Ratio, 95% confidence intervals)*** | | |
| Moderate-Severe HFI | 0.91 (0.69, 1.18) | 1.25 (0.96, 1.62) |
| Marginal HFI | 1.04 (0.77, 1.38) | 1.03 (0.75, 1.41) |
| Food Secure | 1 | 1 |

Adjusted for: child age, maternal ethnicity, maternal education, and number of children in the household, urbanicity, region, and asset index.
